# Supplementary material for: Anti-drug Antibody Responses Impair Prophylaxis Mediated by AAV-Delivered HIV-1 Broadly Neutralizing Antibodies
Source: Mol Ther. 2019 Jan 12;27(3):650–60. doi: 10.1016/j.ymthe.2019.01.004 (PMC6403482; doi:10.1016/j.ymthe.2019.01.004)
Supplement: Document S1. Figures S1–S3 [file mmc1.pdf]

YMTHE, Volume *27*

Supplemental Information

Anti-drug Antibody Responses Impair Prophylaxis Mediated by AAV-Delivered HIV-1 Broadly Neutralizing Antibodies

Matthew R. Gardner, Ina Fetzer, Lisa M. Kattenhorn, Meredith E. Davis-Gardner, Amber S. Zhou, Barnett Alfant, Jesse A. Weber, Hema R. Kondur, Jose M. Martinez-Navio, Sebastian P. Fuchs, Ronald C. Desrosiers, Guangping Gao, Jeffrey D. Lifson, and Michael Farzan

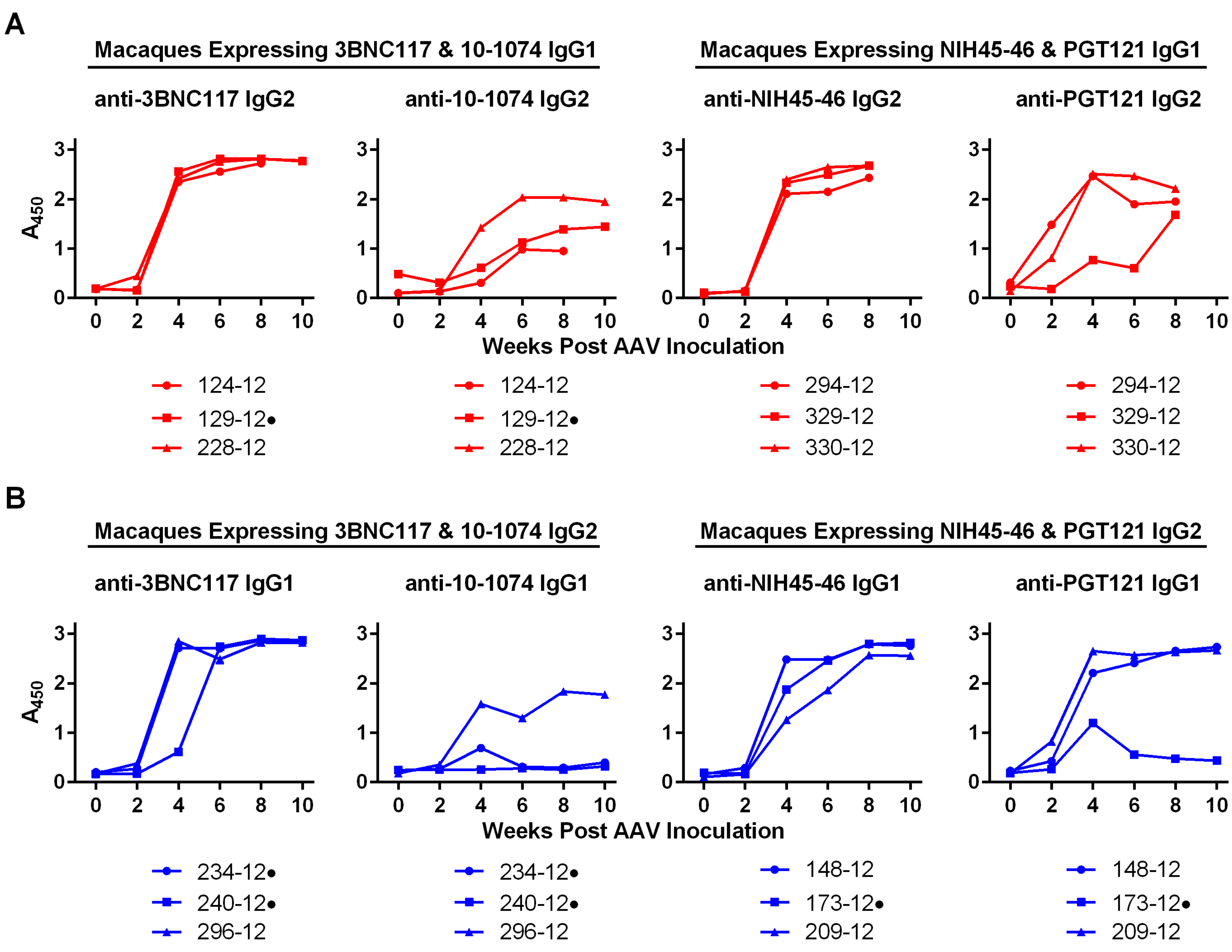

A
Macaques Expressing 3BNC117 & 10-1074 IgG1
Macaques Expressing NIH45-46 & PGT121 IgG1
anti-3BNC117 IgG2
anti-10-1074 IgG2
anti-NIH45-46 IgG2
anti-PGT121 IgG2
A_{450}
Weeks Post AAV Inoculation
124-12
129-12●
228-12
294-12
329-12
330-12
B
Macaques Expressing 3BNC117 & 10-1074 IgG2
Macaques Expressing NIH45-46 & PGT121 IgG2
anti-3BNC117 IgG1
anti-10-1074 IgG1
anti-NIH45-46 IgG1
anti-PGT121 IgG1
A_{450}
Weeks Post AAV Inoculation
234-12●
240-12●
296-12
148-12
173-12●
209-12

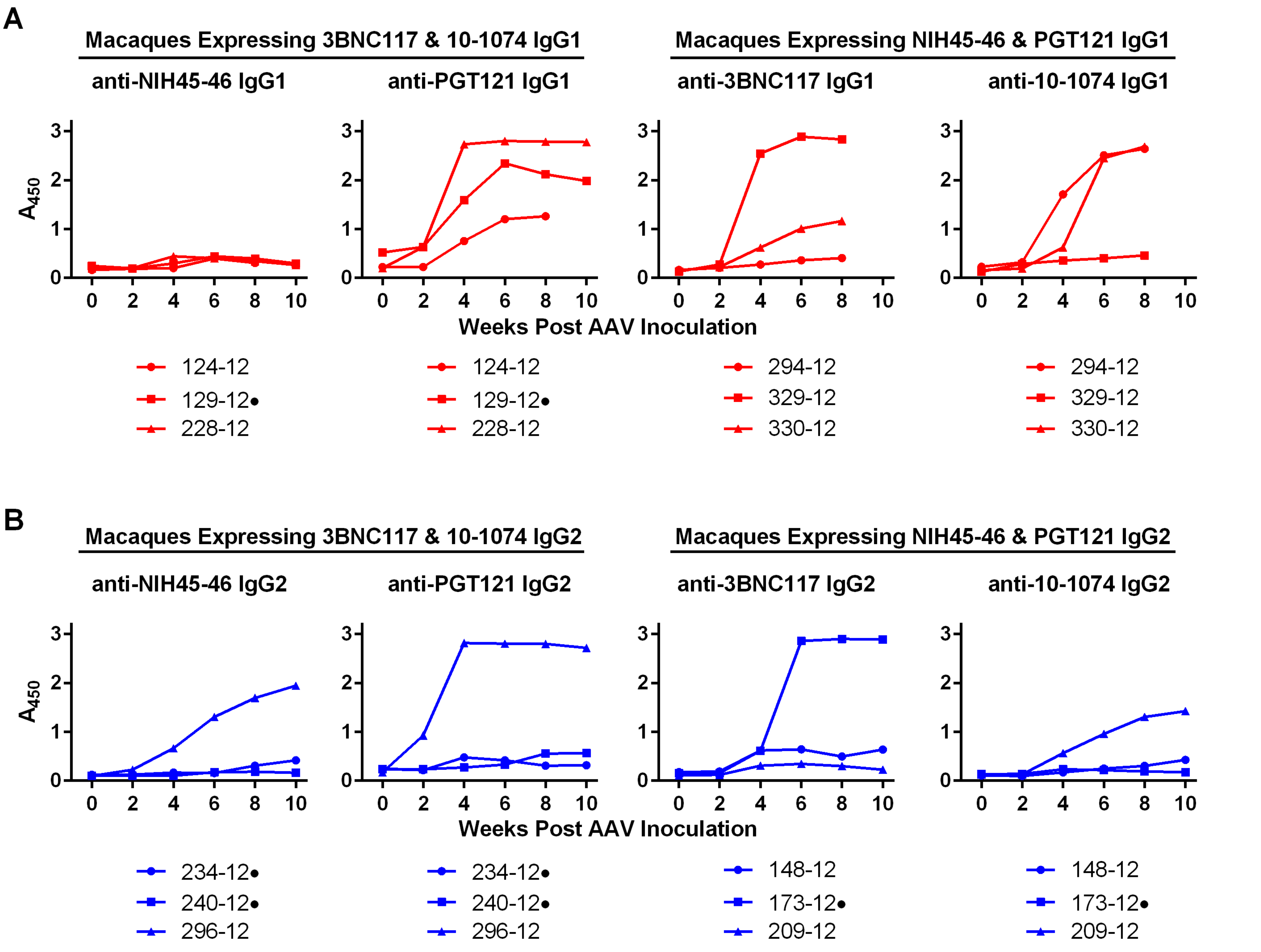

A
Macaques Expressing 3BNC117 & 10-1074 IgG1
Macaques Expressing NIH45-46 & PGT121 IgG1
anti-NIH45-46 IgG1
anti-PGT121 IgG1
anti-3BNC117 IgG1
anti-10-1074 IgG1
A_{450}
Weeks Post AAV Inoculation
124-12
129-12•
228-12
294-12
329-12
330-12
B
Macaques Expressing 3BNC117 & 10-1074 IgG2
Macaques Expressing NIH45-46 & PGT121 IgG2
anti-NIH45-46 IgG2
anti-PGT121 IgG2
anti-3BNC117 IgG2
anti-10-1074 IgG2
234-12•
240-12•
296-12
148-12
173-12•
209-12

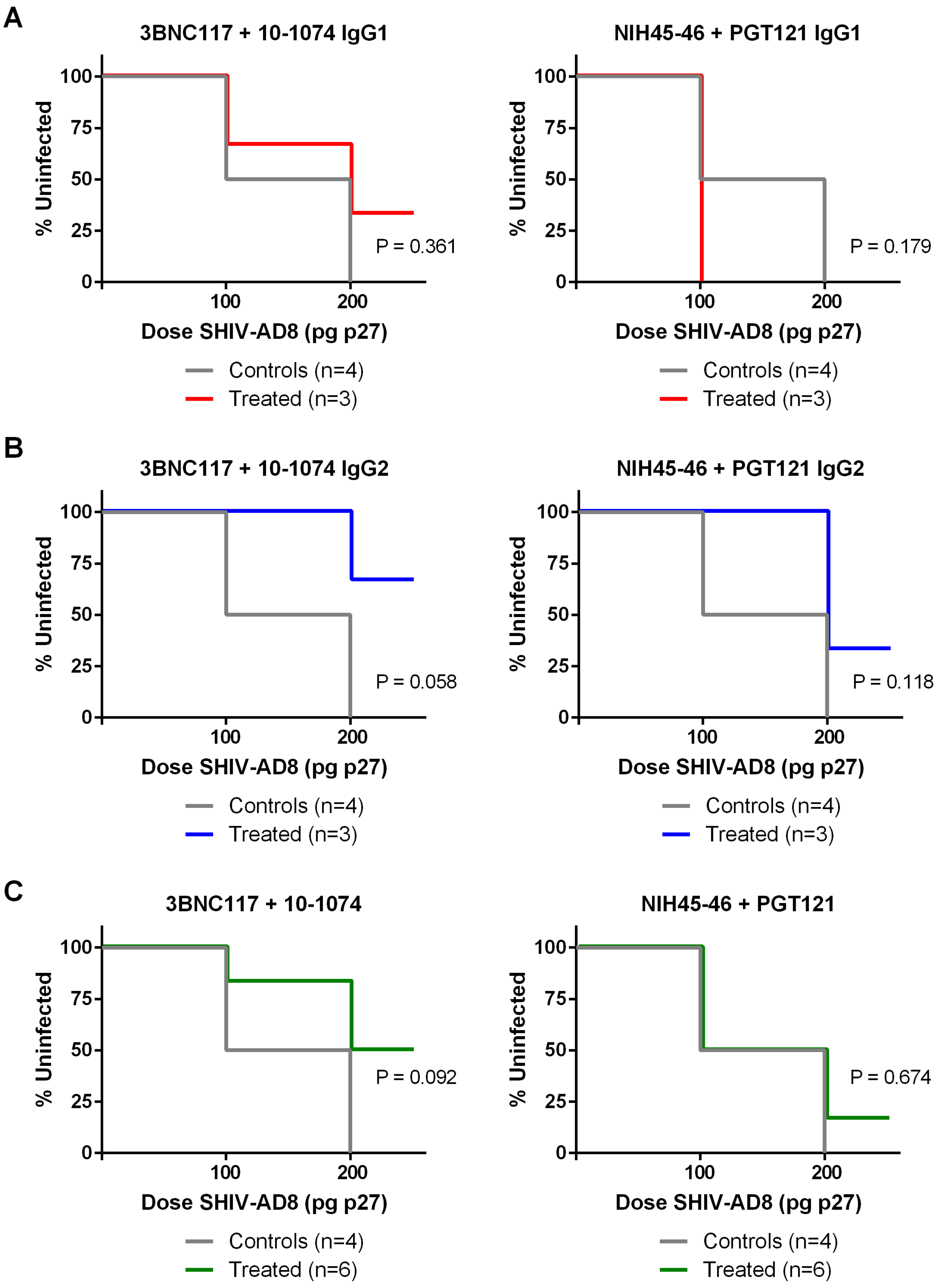
A
3BNC117 + 10-1074 IgG1
% Uninfected
100
75
50
25
0
100
200
Dose SHIV-AD8 (pg p27)
P = 0.361
Controls (n=4)
Treated (n=3)
NIH45-46 + PGT121 IgG1
P = 0.179
Controls (n=4)
Treated (n=3)
B
3BNC117 + 10-1074 IgG2
P = 0.058
Controls (n=4)
Treated (n=3)
NIH45-46 + PGT121 IgG2
P = 0.118
Controls (n=4)
Treated (n=3)
C
3BNC117 + 10-1074
P = 0.092
Controls (n=4)
Treated (n=6)
NIH45-46 + PGT121
P = 0.674
Controls (n=4)
Treated (n=6)

Supplemental Figure Legends

Figure S1. Host ADA responses target bNAb variable regions.

(A-B) Assays similar to those described in Figure 4 except that sera diluted 20-fold from macaques expressing IgG1 (A) or IgG2 (B) antibodies were analyzed using plates coated with their IgG2 and IgG1 counterparts, respectively. Values indicate absorbance at 450 nM. Error bars indicate range of measured values. Dots to the right of animal identification indicate protection from SHIV-AD8, as described in Figure 6.

Figure S2. Cross-reactive ADA to bNAbs within the same class.

(A-B) Assays similar to those described in Figure 4 except that sera diluted 20-fold from macaques expressing IgG1 (A) or IgG2 (B) antibodies were analyzed using plates coated with antibodies different from those which the indicated macaques expressed. The sera from animals expressing 3BNC117 and 10-1074 was analyzed with plates coated with NIH45-46 and PGT121 of the same isotype, and vice versa, as indicated. Values indicate absorbance at 450 nM. Error bars indicate range of measured values. Dots to the right of animal identification indicate protection from SHIV-AD8, as described in Figure 6.

Figure S3. Infection curves for macaques expressing individual bNAb pairs.

(A-B) Kaplan-Meier curves indicating infection of control macaques (grey) or macaques expressing indicated IgG1 (A, red) or IgG2 (B, blue) bNAb pairs, with challenge dose indicated on the horizontal axis. (C) Kaplan-Meier curves indicating infection of control macaques (grey) and indicated bNAb pairs regardless of isotype (green). No significant differences were observed in A-C (Mantel-Cox test).
