# Supplementary material for: Anti-drug Antibody Responses Impair Prophylaxis Mediated by AAV-Delivered HIV-1 Broadly Neutralizing Antibodies
Source: Mol Ther. 2019 Jan 12;27(3):650–60. doi: 10.1016/j.ymthe.2019.01.004 (PMC6403482; doi:10.1016/j.ymthe.2019.01.004)
Supplement: Document S2. Article plus Supplemental Information [file mmc2.pdf]

Molecular Therapy
Original Article

Anti-drug Antibody Responses Impair Prophylaxis Mediated by AAV-Delivered HIV-1 Broadly Neutralizing Antibodies

Matthew R. Gardner,[1] Ina Fetzer,[1] Lisa M. Kattenhorn,[2,7] Meredith E. Davis-Gardner,[1] Amber S. Zhou,[1] Barnett Alfant,[1] Jesse A. Weber,[1] Hema R. Kondur,[1] Jose M. Martinez-Navio,[3] Sebastian P. Fuchs,[3] Ronald C. Desrosiers,[3] Guangping Gao,[4,5] Jeffrey D. Lifson,[6] and Michael Farzan[1]

[1]Department of Immunology and Microbiology, The Scripps Research Institute, Jupiter, FL 33458, USA; [2]Department of Microbiology and Immunobiology, Harvard Medical School, New England Primate Research Center, Southborough, MA 01772, USA; [3]Department of Pathology, University of Miami Miller School of Medicine, Miami, FL 33136, USA; [4]The Horae Gene Therapy Center, University of Massachusetts Medical School, Worcester, MA 01605, USA; [5]Department of Microbiology and Physiological Systems, University of Massachusetts Medical School, Worcester, MA 01605, USA; [6]AIDS and Cancer Virus Program, Frederick National Laboratory for Cancer Research, Frederick, MD 21702, USA

Adeno-associated virus (AAV) delivery of potent and broadly neutralizing antibodies (bNAbs is a promising approach for the prevention of HIV-1 infection. The immunoglobulin G (IgG)1 subtype is usually selected for this application, because it efficiently mediates antibody effector functions and has a somewhat longer half-life. However, the use of IgG1-Fc has been associated with the generation of anti-drug antibodies (ADAs) that correlate with loss of antibody expression. In contrast, we have shown that expression of the antibody-like molecule eCD4-Ig bearing a rhesus IgG2-Fc domain showed reduced immunogenicity and completely protected rhesus macaques from simian-HIV (SHIV)-AD8 challenges. To directly compare the performance of the IgG1-Fc and the IgG2-Fc domains in a prophylactic setting, we compared AAV1 expression of rhesus IgG1 and IgG2 forms of four anti-HIV bNAbs: 3BNC117, NIH45-46, 10-1074, and PGT121. Interestingly, IgG2-isotyped bNAbs elicited significantly lower ADA than their IgG1 counterparts. We also observed significant protection from two SHIV-AD8 challenges in macaques expressing IgG2-isotyped bNAbs, but not from those expressing IgG1. Our data suggest that monoclonal antibodies isotyped with IgG2-Fc domains are less immunogenic than their IgG1 counterparts, and they highlight ADAs as a key barrier to the use of AAV1-expressed bNAbs.

INTRODUCTION

To date, no HIV-1 vaccine approaches have elicited potent broadly neutralizing antibodies (bNAbs) in humans, but bNAbs emerge naturally in some HIV-1-positive individuals after several years of infection (reviewed in Burton and Mascola[1]). These bNAbs target multiple epitopes of the HIV-1 envelope glycoprotein (Env), including the CD4-binding site, the N332 V3-glycan site, the V1V2 apex, the gp120/gp41 interface, and the membrane-proximal external region (MPER). Passive infusion of bNAbs can protect rhesus macaques from infection with simian-HIVs (SHIVs),[2] and it can be used to suppress an established SHIV infection.[3,4] Currently, three bNAbs have been evaluated in clinical trials for safety and therapeutic efficacy.[5–9] Two of these, 3BNC117 and VRC01, target the CD4-binding site, and one, 10-1074, targets sequences that include the N332 glycan at the base of the V3 loop.

One way to bypass the need to develop bNAbs through vaccination is through the use of adeno-associated virus (AAV) vectors. These vectors have been used to express bNAbs and other biologics to prevent HIV-1 or SHIV transmission in animal models. Prophylaxis studies pioneered by Johnson et al.[10] showed that AAV-delivered immunoadhesins could protect most rhesus macaques from an SIVmac316 challenge. Balazs et al.[11,12] extended this approach by showing that AAV8-delivered bNAbs could protect humanized mice from HIV-1. However, more recent macaque studies made it clear that AAV1- and AAV8-delivered bNAbs are frequently targeted by host immune responses, limiting their expression and efficacy.[13–15] To develop an effective AAV-based HIV-1 vaccine alternative, it will, therefore, be necessary to minimize immune system-mediated clearance of the AAV-expressed transgene.

The most effective human HIV-1 vaccine trial to date identified non-neutralizing antibodies recognizing the HIV-1 Env variable domains 1 and 2 as a correlate of protection,[16,17] although this interpretation has been contested.[18] These observations, combined with studies in

Received 19 August 2018; accepted 8 January 2019;
https://doi.org/10.1016/j.ymthe.2019.01.004.

[7]Present address: Novartis Institutes for Biomedical Research, Cambridge, MA, USA.

Correspondence: Matthew R. Gardner, Department of Immunology and Microbiology, The Scripps Research Institute, Jupiter, FL 33458, USA.
E-mail: mgardner@scripps.edu

 © 2019 The Author(s).
This is an open access article under the CC BY license (http://creativecommons.org/licenses/by/4.0/).

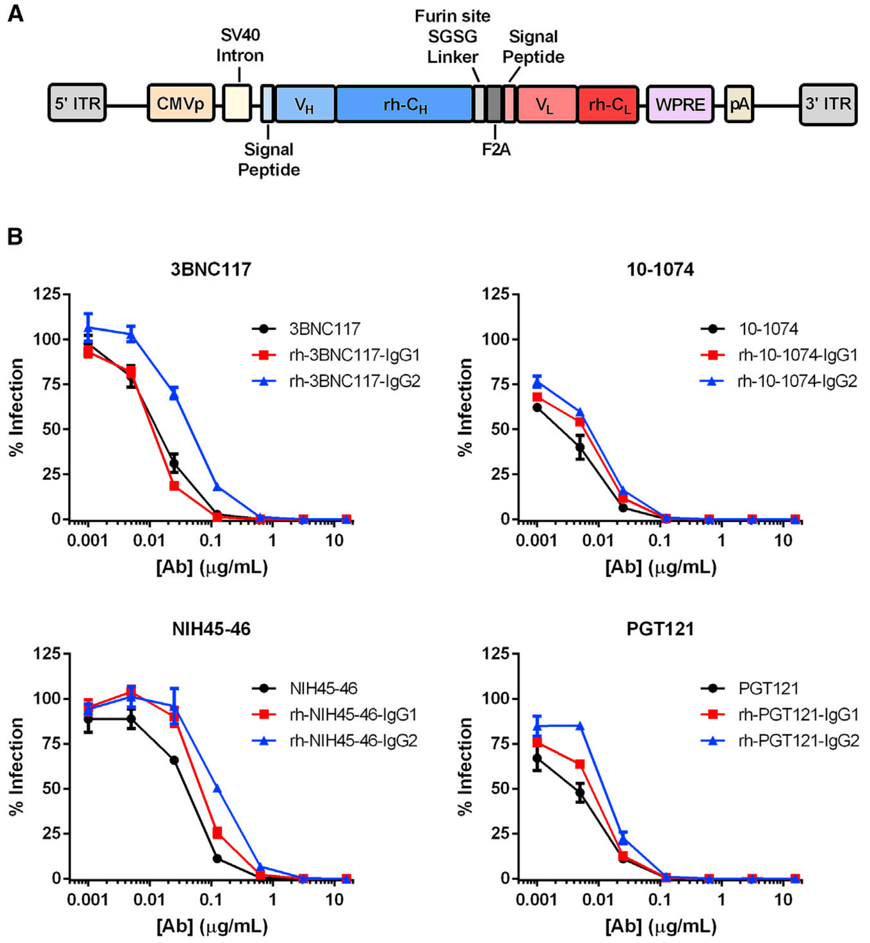

Figure 1. Functional Characterization of HIV-1 bNAbs Expressed from an AAV Gene Cassette

(A) A diagram of the gene cassette used to express bNAbs from an AAV vector. ITR, inverted terminal repeat; CMVp, cytomegalovirus promoter; V_H, heavy-chain variable domain; rh-C_H, rhesus macaque heavy-chain constant domain; V_L, light-chain variable domain; rh-C_L, rhesus macaque light-chain constant domain; WPRE, woodchuck hepatitis virus post-transcriptional regulatory element; pA, SV40 polyadenylation signal sequence. (B) SHIV-AD8 neutralization studies of human IgG1 bNAbs (black) and their rhesus IgG1 (red) and IgG2 (blue) counterparts purified from supernatants of 293T cells transfected with transfer plasmids represented in (A). Error bars indicate SEM.

animal models, raise the possibility that antibody effector functions might play an important role in preventing HIV-1 transmission.[19,20] Thus, antibodies with immunoglobulin G (IgG)1 Fc domains are generally used to study antibody-mediated prophylaxis. In every study of AAV1-delivered bNAbs in rhesus macaques, rhesus IgG1-Fc domains were used, high anti-drug antibody (ADA) responses were observed, and these ADAs limited or abrogated protection.[13–15] Conversely, AAV1-delivered rh-eCD4-Ig that included a rhesus IgG2-Fc domain afforded robust protection from SHIV-AD8 challenges.[21] We thus hypothesized that AAV-delivered bNAbs and eCD4-Ig with IgG2-Fc domains would afford greater protection than their IgG1 counterparts, because the IgG2-Fc may elicit fewer ADAs that limit serum concentrations or activity of the AAV-expressed transgene.

Here we show that macaques inoculated with recombinant AAV1 vectors expressing rhesusized bNAbs with IgG1-Fc domains had high ADAs; low bNAb titers; and, in general, were poorly protected from SHIV-AD8 challenges. However, bNAb combinations with rhesus IgG2-Fc domains elicited fewer ADAs than their IgG1 counterparts, and, partly as a consequence, we observed significant SHIV-AD8 protection in macaques expressing bNAbs with IgG2-Fc domains. Our data suggest that IgG2 forms of bNAbs and other biologics may be useful to limit ADAs in contexts where antibody effector functions are unnecessary or less relevant.

RESULTS

Expression of AAV1-Delivered bNAbs Stabilizes at Low Concentrations

To test the role of isotype in prophylaxis, the variable regions of four bNAbs—PGT121, 10-1074, 3BCN117, and NIH45-46—were combined with rhesus-derived IgG1 or IgG2 constant regions. 3BNC117 and NIH45-46 are CD4-binding site antibodies derived from the same heavy-chain germline-variable gene allele (V_H1-2), but from distinct donors. PGT121 and 10-1074 are N332-glycan antibodies from the same donor.[22] Rhesus IgG1 and IgG2 forms of the heavy chains of these antibodies were cloned into a previously described vector alongside rhesus forms of their respective light chains,[23] as depicted in Figure 1A. The framework regions of bNAb-variable regions were not rhesusized, because these mutations are necessary for bNAb potency.[24] The rhesusized bNAbs expressed from these transfer plasmids neutralized SHIV-AD8 with similar efficiencies, regardless of species-origin or Fc-domain isotype (Figure 1B). The most notable difference was 3BNC117 with a rhesus IgG2-Fc showed to be roughly 5-fold less potent than the human IgG1 3BNC117.

Recombinant AAV1 vectors encoding the rhesusized IgG1 or IgG2 versions of the four bNAbs were then evaluated for expression in NSG (NOD/SCID/IL2R$\gamma^{-/-}$) mice. Eight groups of four mice per group were with 10^{11} total genome copies (GCs) of the AAV1 vectors encoding one of the eight bNAbs (Figure 2A). Concentrations at 4 weeks post-inoculation ranged from 80–143 μg/mL 3BNC117, 33–158 μg/mL 10-1074, 15–58 μg/mL NIH45-46, and 6–47 μg/mL PGT121. In three of four cases, no differences were observed in expression and total average

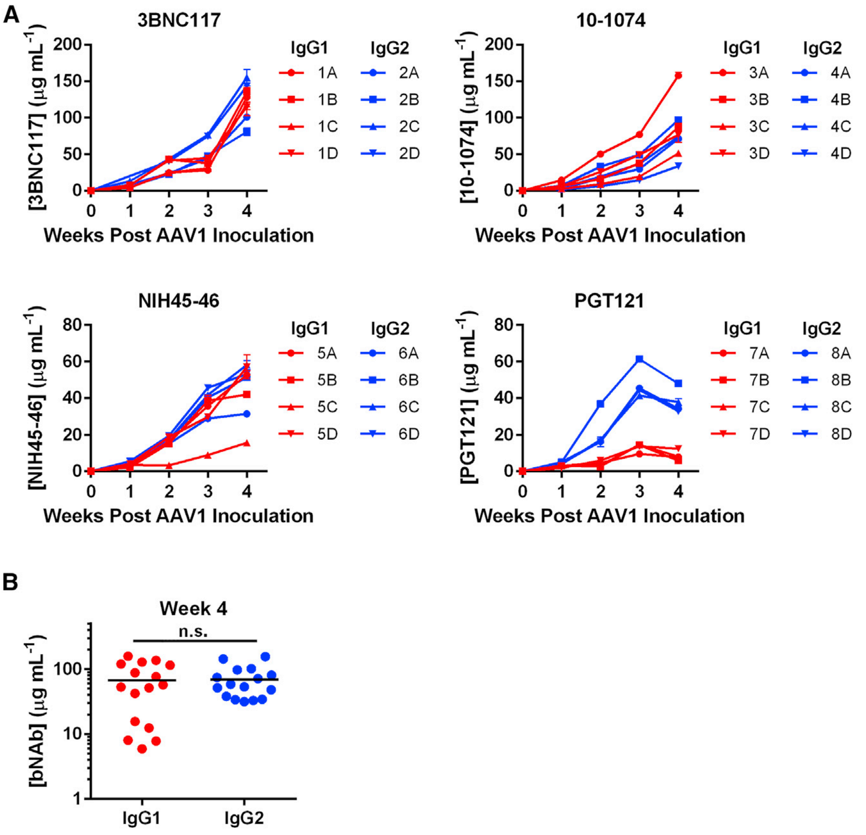

Figure 2. bNAb Expression from AAV1 Vectors in NSG Mice

(A) Concentrations of rhesusized IgG1 (red) or IgG2 (blue) bNAbs in NSG mice. Mice were inoculated with 10^{11} AAV1 vectors encoding the indicated bNAb in the gastrocnemius muscle. Plasma samples were taken from weekly blood draws for 4 weeks, and bNAb concentrations were determined by gp120 ELISA. Error bars indicate the range of measured values. (B) Comparison of bNAb titers from mice expressing IgG1 or IgG2 bNAbs at 4 weeks post-AAV1 inoculation. *n.s., not significant based on unpaired t test.

expression was nearly identical (Figure 2B). However, there was a notable difference in PGT121 IgG2 expression compared to the IgG1 version.

To determine if bNAb expression patterns were similar in mice and macaques, a group of 12 rhesus macaques were inoculated with 10^{13} GCs of a recombinant AAV1 vector encoding either IgG1 or IgG2 forms of one CD4-binding site antibody (3BNC117 or NIH45-46) and the same amount of a vector encoding an N332-glycan antibody (10-1074 or PGT121) of the same isotype. Note that CD4-binding site antibodies used kappa light chains, whereas N332-glycan antibodies used lambda light chains. This feature allowed measurement of both bNAbs in the same animal by using secondary antibodies that reacted with only the kappa or lambda light chain of the bNAb. We observed that bNAb titers peaked at 2–4 weeks, ranging from 3 to 69 μg/mL, and then rapidly declined, in most cases to <1 μg/mL by week 8 (Figures 3A and 3B). Notably, IgG2 bNAbs had significantly greater peak expression than their IgG1 counterparts (Figure 3C; p value of 0.04), but these differences diminished over time (Figure 3D). However, the significance of these peak expression differences disappeared if PGT121 was excluded from this analysis (p value of 0.06). In addition to a trend toward higher expression associated with IgG2, 10-1074 appeared to reach higher levels *in vivo* than did the other antibodies.

Emergence of ADA Coincides with a Decrease in bNAb Expression

Given their low expression, we investigated whether the rapid decline in bNAb titers was associated with the emergence of ADA. ADA was observed in all 12 macaques and against all bNAbs (Figures 4A and 4B). In general, 10-1074 appeared to be less immunogenic than the other antibodies. Notably, in several other cases, ADAs declined to below background levels over the 20-week study, but they remained high in others. Because ADA measurements at week 4 reached the upper limits of the ELISA used and remained high throughout the course of the study, serial dilutions at this time point were performed using the week 4 and week 20 time point samples (Figures 5A and 5B). These dilutions made clear that, over the course of the study, ADAs against IgG1-isotyped bNAbs were significantly greater than those elicited by IgG2 bNAbs (Figures 5C and 5D). Thus, the higher concentrations of IgG2 bNAbs observed in the first several weeks following AAV1 inoculation correlated with lower ADAs in this time frame.

ADA Responses Target the Variable Regions of AAV-Delivered HIV-1 bNAbs

Measured ADAs remained largely consistent whether IgG1 or IgG2 isotypes of each individual bNAb were used to coat the ELISA plate (Figures S1A and S1B), indicating that most ADAs targeted the bNAb-variable regions. We also investigated whether ADA against one bNAb of a class would cross-react with another bNAb from the same class (Figures S2A and S2B). We observed cross-reactivity between the CD4-binding site antibodies 3BNC117 and NIH45-46, which originate from a common variable heavy-chain gene, in four of 12 cases. ADA appeared to be antibody specific, with the exception of some cross-reactivity found in closely related bNAbs. We also observed cross-reactivity between N332-glycan antibodies 10-1074 and PGT121 in seven of 12 cases. These antibodies arose from the same VDJ recombination event. Altogether, our data indicate that most ADAs targeted the bNAb-variable regions and ADA cross-reactivity between related antibodies argues against the use of similar bNAbs in antibody cocktails.

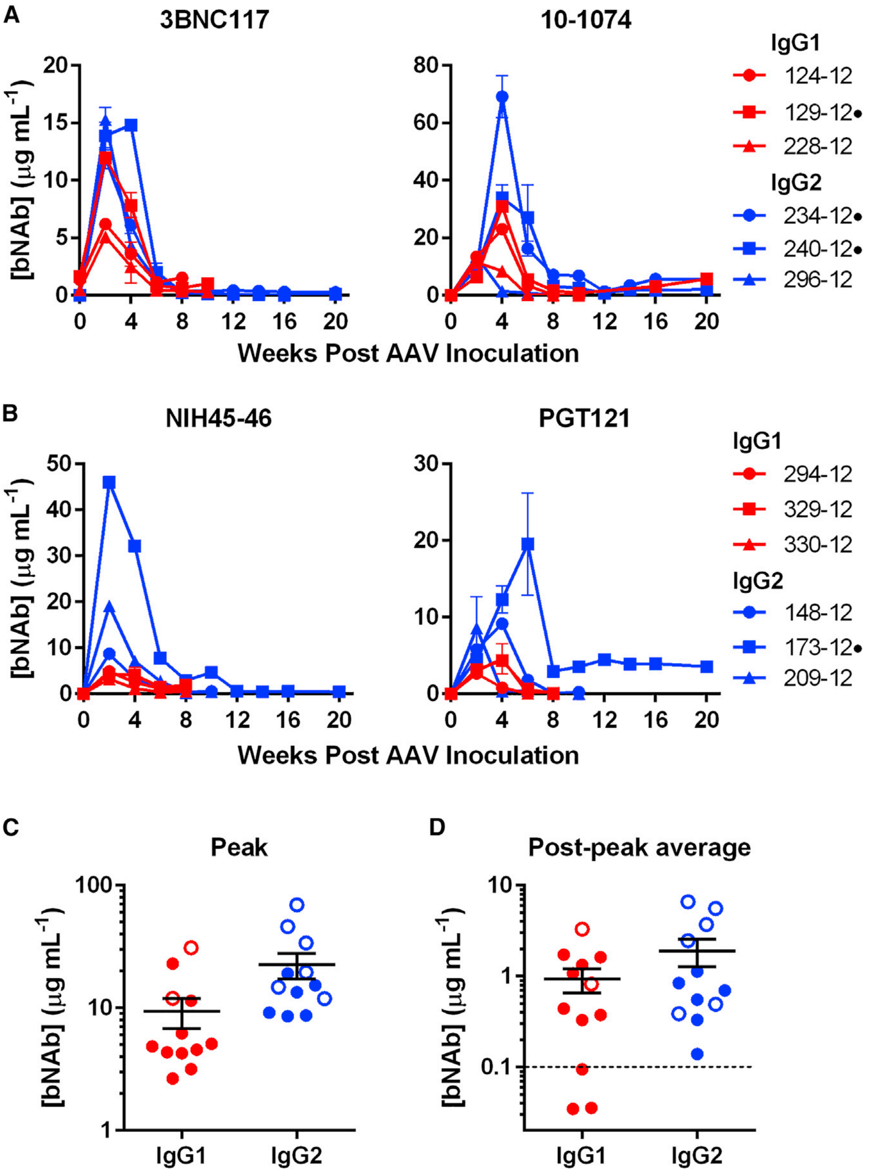

Figure 3. Concentrations of AAV-Delivered HIV-1 bNAbs with Rhesus IgG1 or IgG2 Constant Regions

(A and B) Concentrations of rhesusized IgG1 (A, red) and IgG2 (B, blue) bNAbs in rhesus macaques inoculated intramuscularly with 10^{13} AAV GCs of each indicated antibody. Dots to the right of animal identification number indicate protection from SHIV-AD8, as described in Figure 6. (C and D) Comparison of bNAb titers from macaques expressing IgG1 or IgG2 bNAbs at the peak of expression (C) or the post-peak average (weeks 6–20 post-AAV inoculation) (D). Open circles indicate animals protected from two SHIV-AD8 challenges. Dotted lines indicate limit of detection of the ELISA. Error bars in (A) and (B) indicate range of measured values. Bars in (C) and (D) indicate mean and SD.

Macaques Expressing IgG2-Isotyped, but Not IgG1-Isotyped, bNAbs Are Partially Protected from Low-Dose SHIV-AD8 Challenges

The 12 bNAb-expressing macaques, along with four control animals, were then challenged intravenously with SHIV-AD8.[25] Animals received a 100 pg p27 dose at week 8 post-AAV1 inoculation and a 200 pg dose at week 10 post-AAV1 inoculation (Figure 6A). The challenge doses selected were based on our previous study where a 200-pg p27 dose of this same SHIV-AD8 stock infected 50% of control animals.[21] After the first challenge, none of the animals expressing IgG2 bNAbs were infected. In contrast, 67% of animals expressing IgG1 bNAbs and 50% of the control animals became infected. After the second challenge, 50% of macaques with IgG2 bNAbs, 83% of macaques with IgG1 bNAbs, and all the control macaques were infected. Significant protection was observed with IgG2 bNAbs ($p = 0.032$, Mantel-Cox test), whereas macaques expressing IgG1 bNAbs did not show significant protection relative to the control animals ($p = 0.915$, Mantel-Cox test). No difference in viral loads post-infection were observed in any group (Figure 6B). Differences among antibody pairs were also not significant (Figures S3A–S3C). Protection correlated with lower ADAs against the expressed antibodies at week 4 post-inoculation (Figure 7A) and higher average post-peak expression (weeks 6–20 post-AAV1 inoculation) of an N332-glycan antibody (Figure 7B), but it was independent of CD4-binding site antibody expression (Figure 7C). Thus, lower ADAs and higher bNAb concentrations, associated with AAV-delivered IgG2-bearing bNAbs, correlated with greater protection.

DISCUSSION

All HIV-1 vaccine candidates have so far failed to elicit bNAbs thought necessary for sterilizing protection of the global population. Passively administered bNAbs can effectively prevent HIV-1 transmission, but this approach requires repeated injections for at-risk groups over prolonged periods. In contrast, AAV vectors have the potential to provide a means of durably expressing protective titers of bNAbs or engineered proteins like eCD4-Ig.[21] A key challenge to developing AAV-based vaccine approaches is the problem of ADAs. This problem may have been exacerbated by the human origin of the antibody variable region and by the extensive somatic hypermutation of these bNAbs. Although ADAs have been associated with passively infused antibodies or biologics in humans (reviewed in Krishna and Nadler[26]), they appear less pronounced when a bNAb is passively infused than when it is delivered via AAV.[27] This is likely because the viral vector includes a capsid that is foreign to the host and the viral DNA is likely to stimulate innate immune responses (reviewed

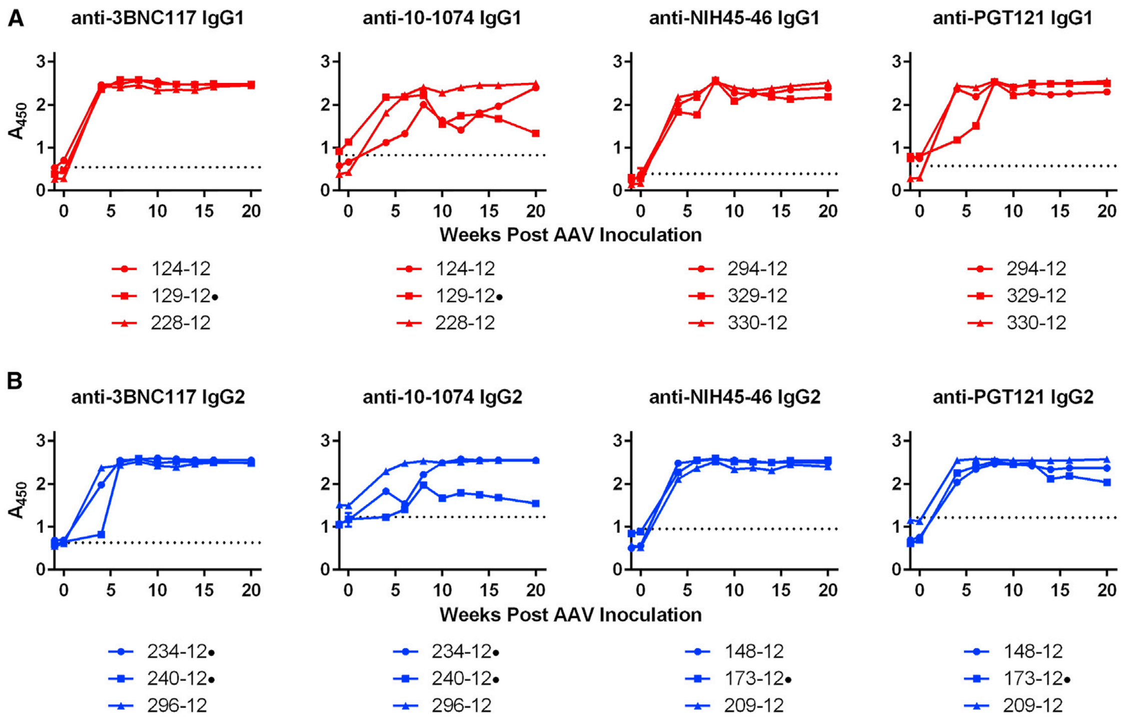

Figure 4. Host ADA Responses against AAV-Expressed IgG1 or IgG2 Versions of HIV-1 bNAbs

(A and B) ADA monitored over the course of 20 weeks in macaques expressing the indicated IgG1 (A, red) or IgG2 (B, blue) bNAbs, as determined by ELISA. Sera samples used for the analysis at the indicated time points were diluted 20-fold. Values indicate absorbance at 450 nM. Error bars indicate range of measured values. Dotted lines indicate the average absorbance value of AAV-negative control samples before and after SHIV-AD8 infection. Dots to the right of animal identification numbers indicate protection from SHIV-AD8, as described in Figure 5.

previously[28–30]). Inflammation at the site of injection may further exacerbate these responses. Thus, muscle-injected AAV, and especially AAV1, appears to function as a potent adjuvant,[31] increasing the likelihood of ADAs and of cell-mediated clearance of transduced cells.[32]

Macaque studies of AAV1-expressed bNAbs, modified with rhesus macaque constant regions, have shown that ADA is indeed a key concern in this context.[13–15] In contrast, we have previously shown that ADAs to rhesus eCD4-Ig were modest and did not impede prophylaxis.[21] Two factors may contribute to this difference. First, eCD4-Ig includes fewer non-self elements than the heavily hypermutated bNAb-variable regions. Second, eCD4-Ig bears a rhesus IgG2-Fc domain, whereas, to date, every primate study of bNAbs utilized rhesus IgG1. Our data here suggest that both factors contribute. Specifically, every bNAb combination tested here elicited ADAs to some extent, regardless of isotype. Of note, 10-1074 appeared to be less immunogenic than the other three bNAbs, even less immunogenic than 3BNC117 expressed in the same animals, consistent with its greater similarity to germline antibody sequences.[13] However, IgG2 bNAbs afforded significant protection compared with controls, although this protection was much less robust than in our previous study with eCD4-IgG2.[21]

The basis for the greater immunogenicity and clearance of IgG1-isotyped proteins remains to be defined. We speculate that antigen-presenting cells more efficiently internalize IgG1 through their Fc receptors, ultimately promoted by ADA production and cytotoxic lymphocyte (CTL)-mediated clearance of transgene-expressing cells. ADAs can also frequently abrogate the activity of passively administered antibodies and biologics used to treat a range of conditions.[33,34] Our data suggest that IgG2 may also help limit these ADAs as well.

The residual immunogenicity of AAV1-delivered bNAbs remains a challenge, especially if administered to a diverse human population. The AAV1 serotype for the vectors used in this study has been shown to be an especially immunogenic AAV serotype, and fewer ADAs have been observed with AAV8, for example.[35] However, high ADAs have been observed with the AAV8-delivered simianized bNAb VRC07, with a rhesus IgG1-Fc domain.[14] Alternatively, targeting the liver for expression of the transgene may also help by inducing tolerance to the transgene itself.[36–38] Ongoing efforts to regulate transgene expression may help to delay expression until after the initial innate response to the vector capsid and DNA abates.[39] It may also be beneficial to explore the use of Toll-like receptor (TLR) 9 antagonists[40,41] and programmed cell death protein 1 (PD-1) agonists[42] as a means of limiting immune responses at the site of inoculation and rapamycin to induce T regulatory cells.[43–45]

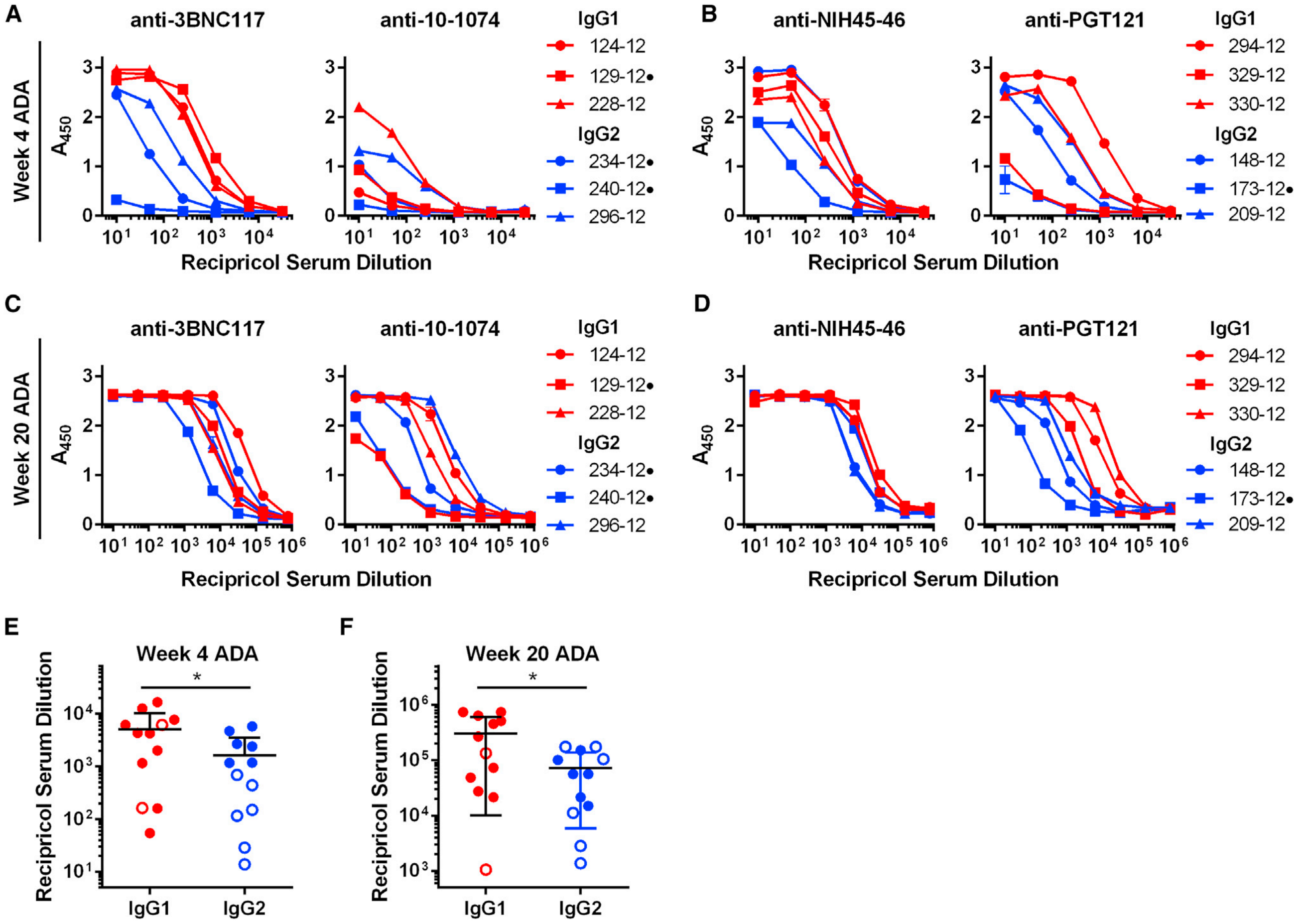

Figure 5. ADA Responses in Macaques Expressing IgG1 and IgG2 bNAbs at 4 and 20 Weeks Post-AAV1 Inoculation

(A–D) Assays similar to those of Figure 3 are shown, except that week 4 and 20 sera samples were serially diluted. Red indicates that sera from macaques expressing the indicated IgG1 bNAbs were analyzed (A), whereas blue indicates that macaques expressing IgG2 bNAbs were analyzed. Dots to the right of animal identification indicate protection from SHIV-AD8, as described in Figure 5. Error bars indicate range of measured values. (E) The reciprocal serum dilution that yields levels of ADA at an $A_{450} = 0.3$ as determined in (A) and (B) is compared. (F) The reciprocal serum dilution that yields levels of ADA at an $A_{450} = 0.3$ as determined in (C) and (D) is compared. Bars indicate mean and SD. *$p < 0.05$, based on unpaired t test.

AAV-expressed anti-HIV inhibitors can serve as an effective vaccine alternative. Unlike current conventional HIV-1 vaccines, this approach requires a one-time inoculation that provides durable protection. The challenge of ADAs will nonetheless be an obstacle that needs to be addressed. In addition, all AAV-delivered antibody approaches require further study of their safety and perhaps including a means of halting transgene expression. Our data shown here indicate that AAV-delivered bNAbs can prevent HIV-1 infection if these challenges are met.

MATERIALS AND METHODS

Non-human Primates, Inoculations, and Challenges

The 16 animals described in this study were Indian-origin rhesus macaques (*Macaca mulatta*), all between 2 and 5 years of age at the time of AAV1 inoculations. All macaques were housed at the New England Primate Research Center in accordance with standards set forth by the American Association for Accreditation of Laboratory Animal Care. This study was performed with the approval of the appropriate Institutional Animal Care and Use Committee (IACUC). All macaques were AAV1 and SIV negative at the beginning of the study. All macaques were negative for Mamu B*08 and B*17 alleles. Macaques that were positive for the Mamu A*01 or A*02 alleles were divided evenly among groups when possible. Macaque ages ranged from 1.92 to 2.25 years old at the time of AAV inoculation. Macaques were inoculated in a quadriceps muscle with 1 mL saline containing 10^{13} AAV1 GCs encoding one CD4-binding site antibody and, in the other quadriceps, 1 mL saline containing 10^{13} AAV1 GCs encoding one N332-glycan antibody. Each quadriceps received two injections of 0.5 mL each. Thus, three macaques received vectors encoding IgG1 forms of 3BNC117 and 10-1074, three macaques received IgG2 forms of these same antibodies, three macaques received IgG1 forms of NIH45-46 and PGT121, and three macaques received

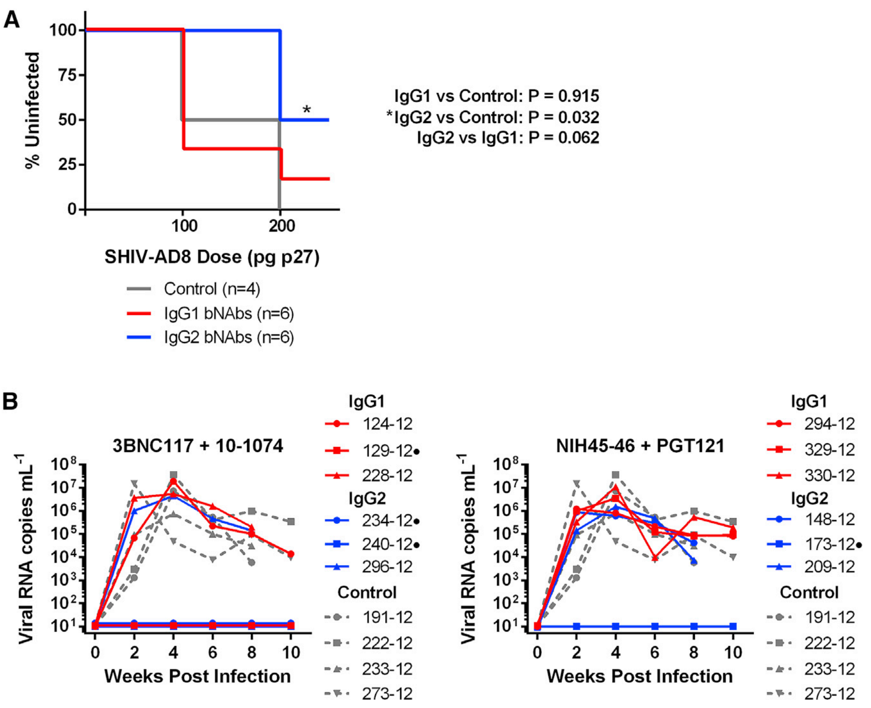

Figure 6. Isotype-Dependent Protection in Macaques Expressing bNAb Pairs

(A) Kaplan-Meier curves indicating infection of control macaques (gray) or macaques expressing IgG1 bNAb pairs (red) or IgG2 bNAb pairs (blue), with intravenous challenge doses at weeks 8 and 10 post AAV-inoculation indicated on the horizontal axis. As shown at the right, significant protection was observed only with IgG2-expressing macaques (Mantel-Cox test). (B) Viral loads of macaques expressing IgG1 (red) or IgG2 (blue) bNAbs aligned at the time of infection. The left panel depicts macaques expressing 3BNC117 and 10-1074. The right panel depicts macaques expressing NIH45-46 and PGT121. Dotted gray lines show viral loads of control macaques. No significant differences in viral loads were observed between any groups. Dots to the right of animal identification indicate protection from SHIV-AD8 after two challenges.

IgG2 forms of these antibodies. 1 mL sera was obtained every 1–2 weeks post-AAV inoculation. Animals were initially challenged with 100 pg p27 SHIV-AD8-EO intravenously. Uninfected animals were re-challenged intravenously 2 weeks later with 200 pg p27 SHIV-AD8-EO intravenously. Plasma viral loads were quantified by RT-PCR as previously described.[3]

Cell Lines and Plasmids

HEK293T cells were obtained from ATCC and grown in DMEM with 10% fetal bovine serum at 37°C. TZM-bl cells were obtained through the NIH AIDS Reagent Program, Division of AIDS, NIAID, NIH from Dr. John C. Kappes, Dr. Xiaoyun Wu, and Tranzyme and grown in DMEM with 10% fetal bovine serum at 37°C.[46–50] The plasmids encoding SHIV-AD8-EO have been previously described.[21] Rhesus macaque IgG1 and IgG2 sequences were based on those described by Scinicariello et al.[51] The rhesus IgG2 isotype versions of 3BNC117, NIH45-46, 10-1074, and PGT121 and the rhesus IgG1 isotype versions of 3BNC117 and 10-1074 have been previously described.[13,21] The rhesus NIH45-46-IgG1 and rhesus PGT121-IgG1 plasmids were generated by cloning a codon-optimized gene (Genewiz, South Plainfield, NJ) into an AAV transfer plasmid containing AAV2 inverted terminal repeats (ITRs) using the NotI-cloning sites. The plasmids encoding the human versions of the heavy and light chains of 3BNC117, NIH45-46, and 10-1074 were previously described.[52] Human PGT121 heavy- and light-chain expression plasmids were a generous gift from Dennis Burton.

Protein Production and Purification

Production of antibodies was performed as previously described.[52] Briefly, HEK293T cells in 175-mm plates were transfected with 80 μg total DNA/plate at 50% confluency with a calcium phosphate transfection kit (Takara, Mountain View, CA). For human antibody production, cells were co-transfected with both heavy-chain vector and light-chain vector at a 1:1 ratio. For rhesus antibody production, cells were co-transfected with AAV transfer plasmid and a plasmid encoding furin at a 4:1 ratio. At 12–16 hr post-transfection, 10% fetal bovine serum (FBS)-DMEM was replaced with serum-free 293 Freestyle media (Invitrogen, Carlsbad, CA). Media were collected after 48 h, and debris was cleared by centrifugation for 10 min at 1,500 × g and filtered using 0.45-μm filter flasks (Millipore Sigma, Billerica, MA). Proteins were isolated with HiTrap columns (GE Healthcare, Pittsburgh, PA) and eluted with IgG Elution Buffer (Thermo Scientific, Waltham, MA) into 1 M Tris-HCl Buffer (pH 9.0) (G Biosciences, St. Louis, MO). Buffer was exchanged with PBS and protein concentrated to 1 mg/mL with Amicon Ultra Centrifugation Filters (Millipore Sigma, Billerica, MA). Antibodies were stored at 4°C.

TZM-bl Neutralization Assay

TZM-bl neutralization assays were performed as previously described.[53] Briefly, SHIV-AD8 was pre-incubated with titrated amounts of antibody in DMEM (10% FBS) for 1 h at 37°C. TZM-bl cells were detached by trypsinization, diluted in DMEM (10% FBS) to 100,000 cells/mL, and added to the virus and inhibitor mixture. Cells were then incubated for 44 h at 37°C. Viral entry was analyzed using Britelite Plus (PerkinElmer, Waltham, MA), and luciferase was measured using a Victor X3 plate reader (PerkinElmer, Waltham, MA).

AAV1 Production and Purification

Production of recombinant AAV1, at the University of Massachusetts Medical School Vector Core, has been previously described.[54] In short, HEK293T cells were transfected with an AAV vector plasmid, a plasmid encoding AAV2 rep and AAV1 cap, and a helper plasmid

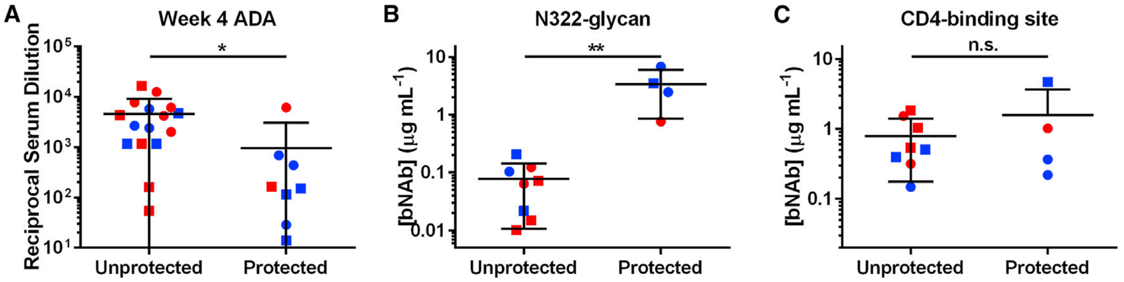

Figure 7. Correlates of SHIV-AD8 Protection by Macaques Expressing AAV-Delivered bNAbs

(A) Comparison of the reciprocal serum dilution that yields background levels of ADA as determined in Figure 3 for macaques that were protected and unprotected from SHIV-AD8 challenges. Red indicates IgG1 and blue indicates IgG2. Circles indicate 3BNC117 or NIH45-46 and squares indicate 10-1074 or PGT121. (B and C) Comparison of bNAb concentrations for N332-glycan bNAbs (B) or CD4-binding site bNAbs (C) for protected and unprotected macaques from SHIV-AD8 challenges. Concentration values are derived from the time point before the macaque became infected or week 10 if protected. Red indicates IgG1 and blue indicates IgG2. Circles indicate 10-1074 (B) or 3BNC117 (C); squares indicate PGT121 (B) or NIH45-46 (C). Bars indicate mean with SD. *$p < 0.05$ and **$p < 0.01$ based on unpaired t test; n.s., not significant.

encoding adenovirus genes. After harvesting lysates of transfected cells, AAV was purified through three sequential cesium chloride (CsCl) centrifugation steps. The vector genome copy number was assessed by RT-PCR. The integrity of AAV particles was verified by electron microscopy (EM), and the purity of the AAV preparations was verified by silver-stained SDS-PAGE.

AAV1 Expression of bNAbs in Mice

32 male NOD.*Cg-Prkdc*^{scid} *IL2rg*^{tm1Wjl}/SzJ mice (NSG/Nod scid gamma) were obtained from The Jackson Laboratory. Mice were inoculated with 10^{11} total genome copies of recombinant AAV1 vectors encoding one of the IgG1 or IgG2 versions of 3BNC117, 10-1074, NIH45-46, or PGT121 at a 25-μL volume in the left gastrocnemius muscle. Mice were bled weekly for 4 weeks, and plasma samples were obtained by centrifuging blood samples at 11,000 $\times$ *g* for 3 min. Plasma was frozen on dry ice and kept at −80°C until used for analysis by gp120 ELISA.

Challenge Virus Production

293T cells were plated in 175-mm flasks and transfected with 80 μg SHIV-AD8-EO by calcium phosphate technique. At 12 h post-transfection, medium was replaced with fresh DMEM (10% FBS). Medium was harvested at 48 h post-transfection, and debris was cleared by centrifugation for 10 min at 1,500 $\times$ *g* and filtered using 0.45-μm filter flasks (Millipore Sigma, Billerica, MA). Virus stocks were aliquoted and frozen at −80°C. Virus titers were determined by an SIV p27 ELISA kit (ABL, Rockville, MD).

gp120 and ADA ELISAs

Half-area 96-well Costar Assay Plates (Corning) were coated with 5 μg/mL gp120 (Immune Tech, New York, NY). Plates were washed with PBS-T (PBS + 0.05% Tween-20) twice and blocked with 5% milk in PBS for 1 h at 37°C. Sera samples were serially diluted in 5% milk in PBS and were added to the plate in duplicate. Standard curves were generated by diluting 4 μg/mL standard protein in 5% milk, then serially diluted and added to the plate in duplicate. Plates were incubated for 1 h at 37°C. Samples were washed five times with PBS-T, and a horseradish peroxidase secondary antibody was added. To determine 3BNC117 and NIH45-46 levels, a secondary antibody recognizing human kappa light chain was used (Southern Biotech, Birmingham, AL). To determine 10-1074 and PGT121 levels, a secondary antibody recognizing human lambda light chain was used (Southern Biotech). Plates were incubated for 1 h at 37°C and then washed ten times with PBS-T. 3,3’,5,5’-Tetramethylbenzidine (TMB) solution (Fisher) was added, and plates were incubated at room temperature until their standard curves developed (typically 5–10 min). TMB Stop Solution (Southern Biotech, Birmingham, AL) was then added and absorbance was read at 450 nm by a Victor X3 plate reader (PerkinElmer, Waltham, MA). Standard curves were analyzed using GraphPad Prism 6.0 software and used to determine protein titers from sera samples.

Sera samples from macaques expressing bNAbs were analyzed against the macaque IgG1 and IgG2 versions of 3BNC117, NIH45-46, 10-1074, and PGT121. Sera samples were diluted 20-fold and blocked in 5% milk in PBS. ADAs from macaques expressing bNAbs were measured using secondary antibodies detecting either kappa or lambda light chains (Southern Biotech, Birmingham, AL) opposite to the antibody being assayed. Plates were incubated for 1 h at 37°C. TMB solution was added for 10 min at room temperature. Stop solution was then added and absorbance at 450 nm was measured as described above.

Ethics Statement

These studies were performed at the New England Primate Research Center, and the statements below apply throughout the time frame of these studies. The animal management program of Harvard Medical School is accredited by the American Association for the Accreditation of Laboratory Animal Care, and it meets NIH standards as set forth in the Guide for the Care and Use of Laboratory Animals (DHHS Publication No. [NIH]85-23 Revised 1985, “The NIH Guide”). The Institute also accepts as mandatory the PHS Policy on Humane Care and Use of Laboratory Animals by Awardee Institutions and NIH Principles for the Utilization and Care of Vertebrate Animals Used in Testing, Research, and Training. There is on file

with the Office for Protection from Research Risks an approved Assurance of Compliance.

Animal facilities met Harvard standards for humane care and use of animals through a program of veterinary care, inspection, and oversight. Animal care and welfare were the charge of the Committee on Animals, appointed by the Dean, consisting of 22 members comprising 4 veterinarians, 2 public representatives, and 16 doctoral-level representatives of principal sites of animal use by Harvard Faculty. Additionally, local facilities were guided and monitored in daily activities by 8 departmental animal use committees. The procedures to avoid unnecessary discomfort, pain, or injury to animals are those prescribed in the aforementioned NIH Guide, and additional detailed protocols for anesthesia, analgesia, tranquilization, euthanasia, or restraint have been developed and circulated by the Committee on Animals.

Rhesus macaques on study were housed in the biocontainment facility of the New England Primate Research Center of Harvard Medical School under approved protocol 04888 of the IACUC of Harvard Medical School. Animals housed in the biocontainment facilities received a daily health check by both animal care technicians and veterinary professional staff. All animals received a complete physical examination on the average of once every 4–6 weeks. A comprehensive environmental enrichment and psychological well-being plan was in place for primates in the described studies, and it is available for inspection by the United States Animal and Plant Health Inspection Service (APHIS) and to officials of any pertinent organization. Euthanasia took place at defined experimental endpoints using protocols consistent with the American Veterinary Medical Association (AVMA) guidelines. Animals were first sedated with intramuscular ketamine hydrochloride at 20 mg/kg body followed by sodium pentobarbital ($\geq$100 mg/kg) intravenously to achieve euthanasia.

Statistical Analyses

All statistical analyses were performed using GraphPad Prism 6.07 software (GraphPad, La Jolla, CA). Comparisons of groups were performed using Student's t test or Mantel-Cox test as reported in the figure legends. Statistical significance is reported as a p value ≤ 0.05.

SUPPLEMENTAL INFORMATION

Supplemental Information includes three figures and can be found with this article online at https://doi.org/10.1016/j.ymthe.2019.01.004.

AUTHOR CONTRIBUTIONS

M.R.G. conceived the study. M.R.G., L.M.K., J.M.M.-N., S.P.F., R.C.D., and M.F. designed the experiments. M.R.G., I.F., L.M.K., M.E.D.-G., A.S.Z., B.A., J.A.W., and H.R.K. performed the experiments. L.M.K. conducted non-human primate studies at NEPRC. M.R.G. and M.E.D.-G. performed the mouse study. G.G. oversaw the production of AAV vectors and screening for AAV1 seropositivity. J.D.L. oversaw viral load quantification experiments. M.R.G., J.D.L., and M.F. analyzed the data. M.R.G. and M.F. wrote the manuscript with input and approval from the other co-authors.

CONFLICTS OF INTEREST

M.R.G. and M.F. are co-founders of Emmune, Inc., a company that has applied for provisional patents for eCD4-Ig and AAV-eCD4-Ig.

ACKNOWLEDGMENTS

The authors would like to thank the staff at the NEPRC for their efforts in handling and performing the required procedures on the animals used in this study and the staff of the Quantitative Molecular Diagnostics Core of the AIDS and Cancer Virus Program, Frederick National Laboratory for Cancer Research for expert technical assistance with viral quantitation assays. The authors would also like to thank Kimberly Kelly for her technical assistance with the mouse study. This project was supported by NIH grants R01 AI080324 (M.F.), UM1 AI126623 (M.F., defeatHIV Delaney Cure Collaboratory), P01 AI100263 (G.G., R.C.D., and M.F.), and F32AI122980 (M.R.G.) and by the Bill and Melinda Gates Foundation OPP1132169 (M.F.). M.R.G. is a recipient of the NIH Loan Repayment Program award. J.D.L. is supported in part with federal funds from the National Cancer Institute, NIH, under contract HHSN261200800001E. The content of this publication does not necessarily reflect the views or policies of the Department of Health and Human Services or the NIH, nor does mention of trade names, commercial products, or organizations imply endorsement by the U.S. government.

REFERENCES

1. Burton, D.R., and Mascola, J.R. (2015). Antibody responses to envelope glycoproteins in HIV-1 infection. Nat. Immunol. *16*, 571–576.
2. Gautam, R., Nishimura, Y., Pegu, A., Nason, M.C., Klein, F., Gazumyan, A., Golijanin, J., Buckler-White, A., Sadjadpour, R., Wang, K., et al. (2016). A single injection of anti-HIV-1 antibodies protects against repeated SHIV challenges. Nature *533*, 105–109.
3. Shingai, M., Nishimura, Y., Klein, F., Mouquet, H., Donau, O.K., Plishka, R., Buckler-White, A., Seaman, M., Piatak, M., Jr., Lifson, J.D., et al. (2013). Antibody-mediated immunotherapy of macaques chronically infected with SHIV suppresses viraemia. Nature *503*, 277–280.
4. Barouch, D.H., Whitney, J.B., Moldt, B., Klein, F., Oliveira, T.Y., Liu, J., Stephenson, K.E., Chang, H.W., Shekhar, K., Gupta, S., et al. (2013). Therapeutic efficacy of potent neutralizing HIV-1-specific monoclonal antibodies in SHIV-infected rhesus monkeys. Nature *503*, 224–228.
5. Caskey, M., Schoofs, T., Gruell, H., Settler, A., Karagounis, T., Kreider, E.F., Murrell, B., Pfeifer, N., Nogueira, L., Oliveira, T.Y., et al. (2017). Antibody 10-1074 suppresses viremia in HIV-1-infected individuals. Nat. Med. *23*, 185–191.
6. Scheid, J.F., Horwitz, J.A., Bar-On, Y., Kreider, E.F., Lu, C.-L., Lorenzi, J.C.C., Feldmann, A., Braunschweig, M., Nogueira, L., Oliveira, T., et al. (2016). HIV-1 antibody 3BNC117 suppresses viral rebound in humans during treatment interruption. Nature *535*, 556–560.
7. Bar, K.J., Sneller, M.C., Harrison, L.J., Justement, J.S., Overton, E.T., Petrone, M.E., Salantes, D.B., Seamon, C.A., Scheinfeld, B., Kwan, R.W., et al. (2016). Effect of HIV Antibody VRC01 on Viral Rebound after Treatment Interruption. N. Engl. J. Med. *375*, 2037–2050.
8. Lynch, R.M., Boritz, E., Coates, E.E., DeZure, A., Madden, P., Costner, P., Enama, M.E., Plummer, S., Holman, L., Hendel, C.S., et al.; VRC 601 Study Team (2015). Virologic effects of broadly neutralizing antibody VRC01 administration during chronic HIV-1 infection. Sci. Transl. Med. *7*, 319ra206.

9. Ledgerwood, J.E., Coates, E.E., Yamshchikov, G., Saunders, J.G., Holman, L., Enama, M.E., DeZure, A., Lynch, R.M., Gordon, I., Plummer, S., et al.; VRC 602 Study Team (2015). Safety, pharmacokinetics and neutralization of the broadly neutralizing HIV-1 human monoclonal antibody VRC01 in healthy adults. Clin. Exp. Immunol. *182*, 289–301.

10. Johnson, P.R., Schnepp, B.C., Zhang, J., Connell, M.J., Greene, S.M., Yuste, E., Desrosiers, R.C., and Clark, K.R. (2009). Vector-mediated gene transfer engenders long-lived neutralizing activity and protection against SIV infection in monkeys. Nat. Med. *15*, 901–906.

11. Balazs, A.B., Ouyang, Y., Hong, C.M., Chen, J., Nguyen, S.M., Rao, D.S., An, D.S., and Baltimore, D. (2014). Vectored immunoprophylaxis protects humanized mice from mucosal HIV transmission. Nat. Med. *20*, 296–300.

12. Balazs, A.B., Chen, J., Hong, C.M., Rao, D.S., Yang, L., and Baltimore, D. (2011). Antibody-based protection against HIV infection by vectored immunoprophylaxis. Nature *481*, 81–84.

13. Martinez-Navio, J.M., Fuchs, S.P., Pedreño-López, S., Rakasz, E.G., Gao, G., and Desrosiers, R.C. (2016). Host Anti-antibody Responses Following Adeno-associated Virus-mediated Delivery of Antibodies Against HIV and SIV in Rhesus Monkeys. Mol. Ther. *24*, 76–86.

14. Saunders, K.O., Wang, L., Joyce, M.G., Yang, Z.-Y., Balazs, A.B., Cheng, C., Ko, S.Y., Kong, W.P., Rudicell, R.S., Georgiev, I.S., et al. (2015). Broadly Neutralizing Human Immunodeficiency Virus Type 1 Antibody Gene Transfer Protects Nonhuman Primates from Mucosal Simian-Human Immunodeficiency Virus Infection. J. Virol. *89*, 8334–8345.

15. Fuchs, S.P., Martinez-Navio, J.M., Piatak, M., Jr., Lifson, J.D., Gao, G., and Desrosiers, R.C. (2015). AAV-Delivered Antibody Mediates Significant Protective Effects against SIVmac239 Challenge in the Absence of Neutralizing Activity. PLoS Pathog. *11*, e1005090.

16. Haynes, B.F., Gilbert, P.B., McElrath, M.J., Zolla-Pazner, S., Tomaras, G.D., Alam, S.M., Evans, D.T., Montefiori, D.C., Karnasuta, C., Sutthent, R., et al. (2012). Immune-correlates analysis of an HIV-1 vaccine efficacy trial. N. Engl. J. Med. *366*, 1275–1286.

17. Bonsignori, M., Pollara, J., Moody, M.A., Alpert, M.D., Chen, X., Hwang, K.-K., Gilbert, P.B., Huang, Y., Gurley, T.C., Kozink, D.M., et al. (2012). Antibody-dependent cellular cytotoxicity-mediating antibodies from an HIV-1 vaccine efficacy trial target multiple epitopes and preferentially use the VH1 gene family. J. Virol. *86*, 11521–11532.

18. Desrosiers, R.C. (2017). Protection against HIV Acquisition in the RV144 Trial. J. Virol. *91*, e00905–e00917.

19. Bournazos, S., Klein, F., Pietzsch, J., Seaman, M.S., Nussenzweig, M.C., and Ravetch, J.V. (2014). Broadly neutralizing anti-HIV-1 antibodies require Fc effector functions for in vivo activity. Cell *158*, 1243–1253.

20. Hessell, A.J., Hangartner, L., Hunter, M., Havenith, C.E., Beurskens, F.J., Bakker, J.M., Lanigan, C.M., Landucci, G., Forthal, D.N., Parren, P.W., et al. (2007). Fc receptor but not complement binding is important in antibody protection against HIV. Nature *449*, 101–104.

21. Gardner, M.R., Kattenhorn, L.M., Kondur, H.R., von Schaewen, M., Dorfman, T., Chiang, J.J., Haworth, K.G., Decker, J.M., Alpert, M.D., Bailey, C.C., et al. (2015). AAV-expressed eCD4-Ig provides durable protection from multiple SHIV challenges. Nature *519*, 87–91.

22. Mouquet, H., Scharf, L., Euler, Z., Liu, Y., Eden, C., Scheid, J.F., Halper-Stromberg, A., Gnanapragasam, P.N., Spencer, D.I., Seaman, M.S., et al. (2012). Complex-type N-glycan recognition by potent broadly neutralizing HIV antibodies. Proc. Natl. Acad. Sci. USA *109*, E3268–E3277.

23. Fuchs, S.P., Martinez-Navio, J.M., Gao, G., and Desrosiers, R.C. (2016). Recombinant AAV Vectors for Enhanced Expression of Authentic IgG. PLoS ONE *11*, e0158009.

24. Klein, F., Diskin, R., Scheid, J.F., Gaebler, C., Mouquet, H., Georgiev, I.S., Pancera, M., Zhou, T., Incesu, R.B., Fu, B.Z., et al. (2013). Somatic mutations of the immunoglobulin framework are generally required for broad and potent HIV-1 neutralization. Cell *153*, 126–138.

25. Nishimura, Y., Shingai, M., Willey, R., Sadjadpour, R., Lee, W.R., Brown, C.R., Brenchley, J.M., Buckler-White, A., Petros, R., Eckhaus, M., et al. (2010). Generation of the pathogenic R5-tropic simian/human immunodeficiency virus SHIVAD8 by serial passaging in rhesus macaques. J. Virol. *84*, 4769–4781.

26. Krishna, M., and Nadler, S.G. (2016). Immunogenicity to Biotherapeutics - The Role of Anti-drug Immune Complexes. Front. Immunol. *7*, 21.

27. Gaudinski, M.R., Coates, E.E., Houser, K.V., Chen, G.L., Yamshchikov, G., Saunders, J.G., Holman, L.A., Gordon, I., Plummer, S., Hendel, C.S., et al.; VRC 606 Study Team (2018). Safety and pharmacokinetics of the Fc-modified HIV-1 human monoclonal antibody VRC01LS: A Phase 1 open-label clinical trial in healthy adults. PLoS Med. *15*, e1002493.

28. Gardner, M.R., and Farzan, M. (2017). Engineering antibody-like inhibitors to prevent and treat HIV-1 infection. Curr. Opin. HIV AIDS *12*, 294–301.

29. Fuchs, S.P., and Desrosiers, R.C. (2016). Promise and problems associated with the use of recombinant AAV for the delivery of anti-HIV antibodies. Mol. Ther. Methods Clin. Dev. *3*, 16068.

30. Mingozzi, F., and High, K.A. (2013). Immune responses to AAV vectors: overcoming barriers to successful gene therapy. Blood *122*, 23–36.

31. Wang, L., Dobrzynski, E., Schlachterman, A., Cao, O., and Herzog, R.W. (2005). Systemic protein delivery by muscle-gene transfer is limited by a local immune response. Blood *105*, 4226–4234.

32. Martino, A.T., Basner-Tschakarjan, E., Markusic, D.M., Finn, J.D., Hinderer, C., Zhou, S., Ostrov, D.A., Srivastava, A., Ertl, H.C., Terhorst, C., et al. (2013). Engineered AAV vector minimizes in vivo targeting of transduced hepatocytes by capsid-specific CD8+ T cells. Blood *121*, 2224–2233.

33. Hoshino, M., Yoshio, T., Onishi, S., and Minota, S. (2012). Influence of antibodies against infliximab and etanercept on the treatment effectiveness of these agents in Japanese patients with rheumatoid arthritis. Mod. Rheumatol. *22*, 532–540.

34. Emi Aikawa, N., de Carvalho, J.F., Artur Almeida Silva, C., and Bonfá, E. (2010). Immunogenicity of Anti-TNF-α agents in autoimmune diseases. Clin. Rev. Allergy Immunol. *38*, 82–89.

35. Lu, Y., and Song, S. (2009). Distinct immune responses to transgene products from rAAV1 and rAAV8 vectors. Proc. Natl. Acad. Sci. USA *106*, 17158–17162.

36. Mingozzi, F., Liu, Y.-L., Dobrzynski, E., Kaufhold, A., Liu, J.H., Wang, Y., Arruda, V.R., High, K.A., and Herzog, R.W. (2003). Induction of immune tolerance to coagulation factor IX antigen by in vivo hepatic gene transfer. J. Clin. Invest. *111*, 1347–1356.

37. Ziegler, R.J., Lonning, S.M., Armentano, D., Li, C., Souza, D.W., Cherry, M., Ford, C., Barbon, C.M., Desnick, R.J., Gao, G., et al. (2004). AAV2 vector harboring a liver-restricted promoter facilitates sustained expression of therapeutic levels of α-galactosidase A and the induction of immune tolerance in Fabry mice. Mol. Ther. *9*, 231–240.

38. Dobrzynski, E., Mingozzi, F., Liu, Y.-L., Bendo, E., Cao, O., Wang, L., and Herzog, R.W. (2004). Induction of antigen-specific CD4+ T-cell anergy and deletion by in vivo viral gene transfer. Blood *104*, 969–977.

39. Zhong, G., Wang, H., Bailey, C.C., Gao, G., and Farzan, M. (2016). Rational design of aptazyme riboswitches for efficient control of gene expression in mammalian cells. eLife *5*, e18858.

40. Martino, A.T., Suzuki, M., Markusic, D.M., Zolotukhin, I., Ryals, R.C., Moghimi, B., Ertl, H.C., Muruve, D.A., Lee, B., and Herzog, R.W. (2011). The genome of self-complementary adeno-associated viral vectors increases Toll-like receptor 9-dependent innate immune responses in the liver. Blood *117*, 6459–6468.

41. Zhu, J., Huang, X., and Yang, Y. (2009). The TLR9-MyD88 pathway is critical for adaptive immune responses to adeno-associated virus gene therapy vectors in mice. J. Clin. Invest. *119*, 2388–2398.

42. Adriouch, S., Franck, E., Drouot, L., Bonneau, C., Jolinon, N., Salvetti, A., and Boyer, O. (2011). Improved Immunological Tolerance Following Combination Therapy with CTLA-4/Ig and AAV-Mediated PD-L1/2 Muscle Gene Transfer. Front. Microbiol. *2*, 199.

43. Biswas, M., Sarkar, D., Kumar, S.R.P., Nayak, S., Rogers, G.L., Markusic, D.M., Liao, G., Terhorst, C., and Herzog, R.W. (2015). Synergy between rapamycin and FLT3 ligand enhances plasmacytoid dendritic cell-dependent induction of $CD4^+CD25^+FoxP3^+$ Treg. Blood *125*, 2937–2947.

44. Mueller, C., Chulay, J.D., Trapnell, B.C., Humphries, M., Carey, B., Sandhaus, R.A., McElvaney, N.G., Messina, L., Tang, Q., Rouhani, F.N., et al. (2013). Human Treg responses allow sustained recombinant adeno-associated virus-mediated transgene expression. J. Clin. Invest. *123*, 5310–5318.

45. Nayak, S., Cao, O., Hoffman, B.E., Cooper, M., Zhou, S., Atkinson, M.A., and Herzog, R.W. (2009). Prophylactic immune tolerance induced by changing the ratio of antigen-specific effector to regulatory T cells. J. Thromb. Haemost. *7*, 1523–1532.

46. Platt, E.J., Bilska, M., Kozak, S.L., Kabat, D., and Montefiori, D.C. (2009). Evidence that ecotropic murine leukemia virus contamination in TZM-bl cells does not affect the outcome of neutralizing antibody assays with human immunodeficiency virus type 1. J. Virol. *83*, 8289–8292.

47. Takeuchi, Y., McClure, M.O., and Pizzato, M. (2008). Identification of gammaretroviruses constitutively released from cell lines used for human immunodeficiency virus research. J. Virol. *82*, 12585–12588.

48. Wei, X., Decker, J.M., Liu, H., Zhang, Z., Arani, R.B., Kilby, J.M., Saag, M.S., Wu, X., Shaw, G.M., and Kappes, J.C. (2002). Emergence of resistant human immunodeficiency virus type 1 in patients receiving fusion inhibitor (T-20) monotherapy. Antimicrob. Agents Chemother. *46*, 1896–1905.

49. Derdeyn, C.A., Decker, J.M., Sfakianos, J.N., Wu, X., O'Brien, W.A., Ratner, L., Kappes, J.C., Shaw, G.M., and Hunter, E. (2000). Sensitivity of human immunodeficiency virus type 1 to the fusion inhibitor T-20 is modulated by coreceptor specificity defined by the V3 loop of gp120. J. Virol. *74*, 8358–8367.

50. Platt, E.J., Wehrly, K., Kuhmann, S.E., Chesebro, B., and Kabat, D. (1998). Effects of CCR5 and CD4 cell surface concentrations on infections by macrophagetropic isolates of human immunodeficiency virus type 1. J. Virol. *72*, 2855–2864.

51. Scinicariello, F., Engleman, C.N., Jayashankar, L., McClure, H.M., and Attanasio, R. (2004). Rhesus macaque antibody molecules: sequences and heterogeneity of alpha and gamma constant regions. Immunology *111*, 66–74.

52. Gardner, M.R., Fellinger, C.H., Prasad, N.R., Zhou, A.S., Kondur, H.R., Joshi, V.R., Quinlan, B.D., and Farzan, M. (2016). CD4-Induced Antibodies Promote Association of the HIV-1 Envelope Glycoprotein with CD4-Binding Site Antibodies. J. Virol. *90*, 7822–7832.

53. Li, M., Gao, F., Mascola, J.R., Stamatatos, L., Polonis, V.R., Koutsoukos, M., Voss, G., Goepfert, P., Gilbert, P., Greene, K.M., et al. (2005). Human immunodeficiency virus type 1 env clones from acute and early subtype B infections for standardized assessments of vaccine-elicited neutralizing antibodies. J. Virol. *79*, 10108–10125.

54. Mueller, C., Ratner, D., Zhong, L., Esteves-Sena, M., and Gao, G. (2012). Production and discovery of novel recombinant adeno-associated viral vectors. Current Protoc. Microbiol. *Chapter 14*, Unit 14D.1.

YMTHE, Volume *27*

Supplemental Information

Anti-drug Antibody Responses Impair Prophylaxis Mediated by AAV-Delivered HIV-1 Broadly Neutralizing Antibodies

Matthew R. Gardner, Ina Fetzer, Lisa M. Kattenhorn, Meredith E. Davis-Gardner, Amber S. Zhou, Barnett Alfant, Jesse A. Weber, Hema R. Kondur, Jose M. Martinez-Navio, Sebastian P. Fuchs, Ronald C. Desrosiers, Guangping Gao, Jeffrey D. Lifson, and Michael Farzan

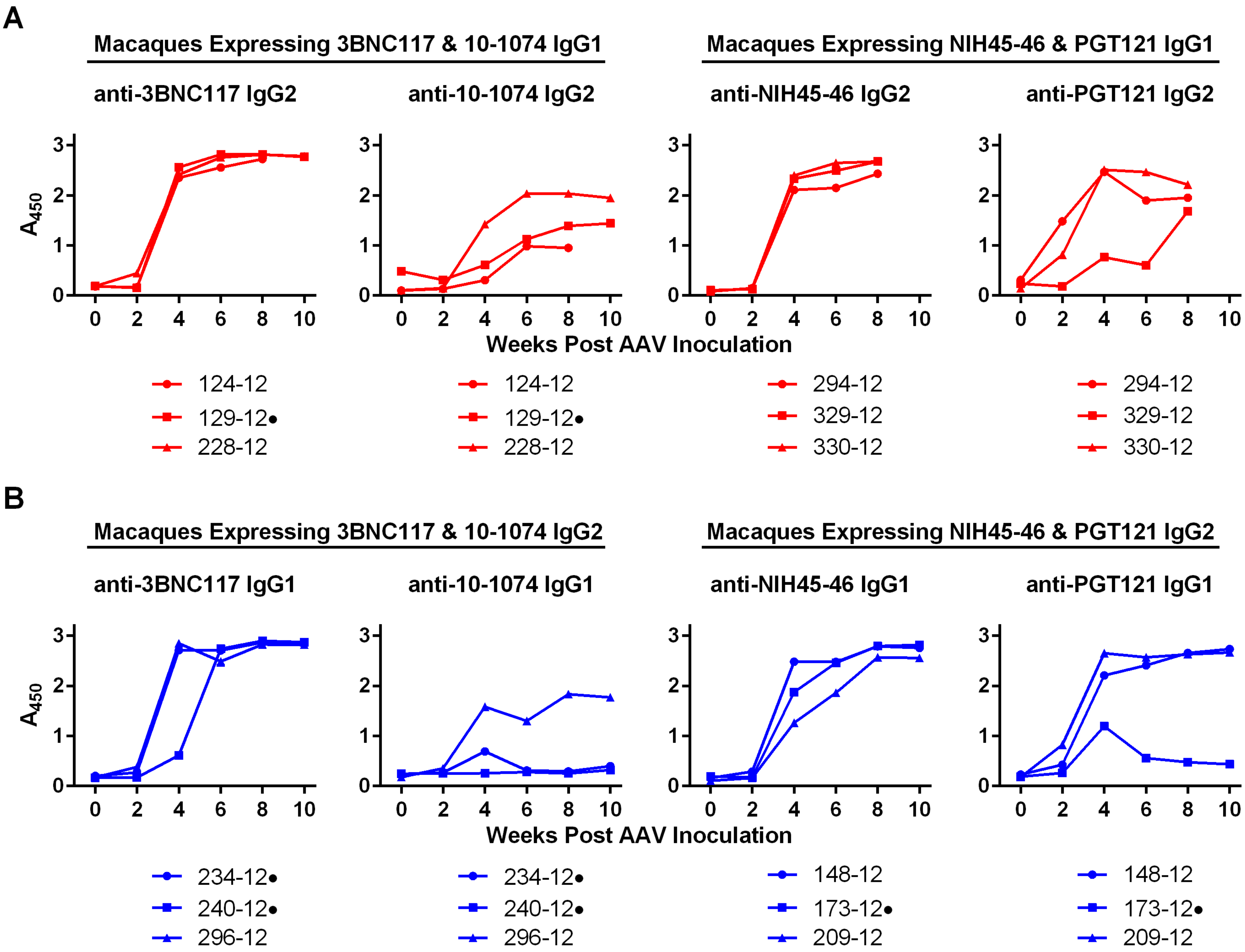
A
Macaques Expressing 3BNC117 & 10-1074 IgG1
Macaques Expressing NIH45-46 & PGT121 IgG1
anti-3BNC117 IgG2
anti-10-1074 IgG2
anti-NIH45-46 IgG2
anti-PGT121 IgG2
A450
Weeks Post AAV Inoculation
124-12
129-12•
228-12
294-12
329-12
330-12
B
Macaques Expressing 3BNC117 & 10-1074 IgG2
Macaques Expressing NIH45-46 & PGT121 IgG2
anti-3BNC117 IgG1
anti-10-1074 IgG1
anti-NIH45-46 IgG1
anti-PGT121 IgG1
A450
Weeks Post AAV Inoculation
234-12•
240-12•
296-12
148-12
173-12•
209-12

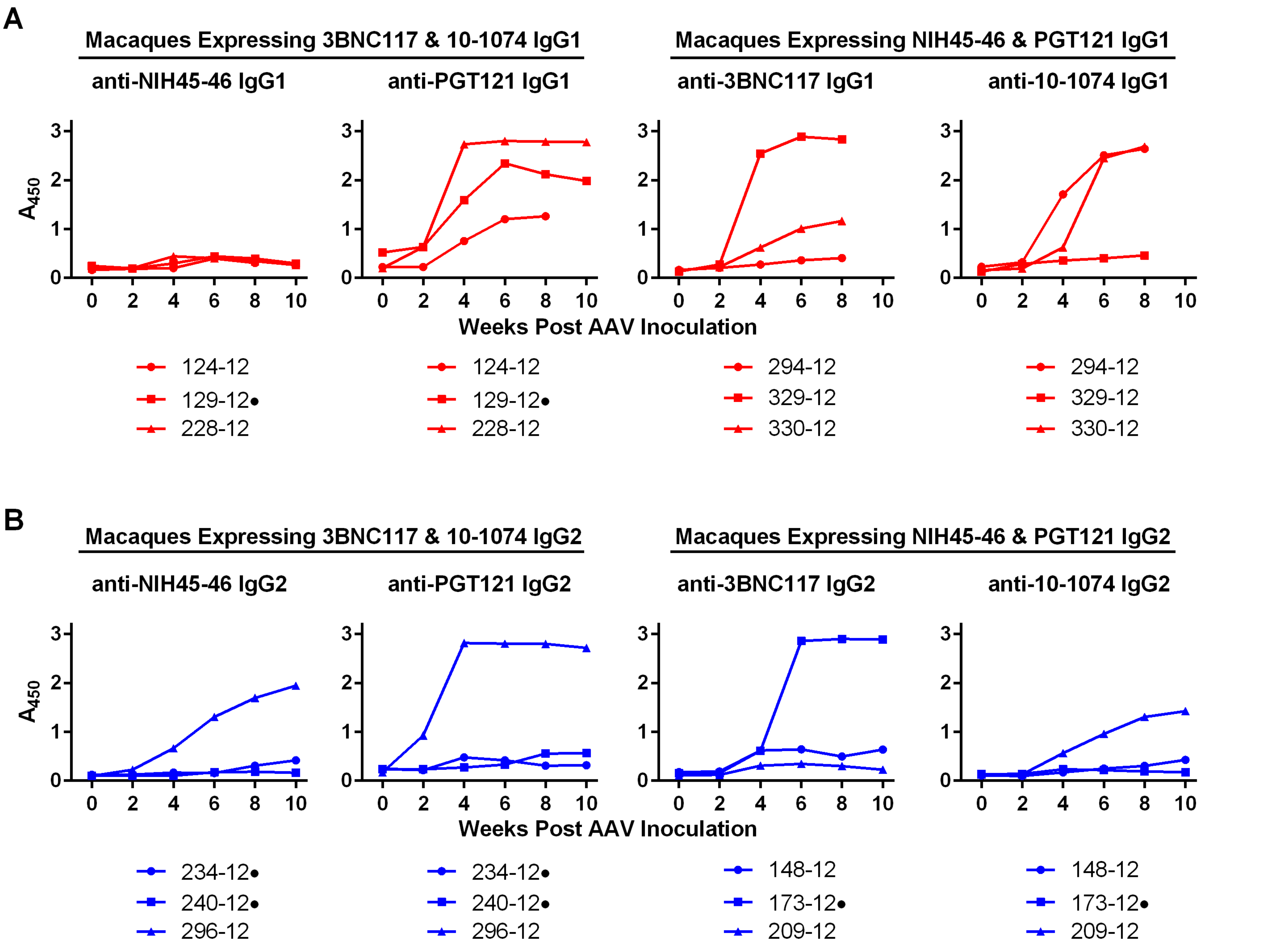
A
Macaques Expressing 3BNC117 & 10-1074 IgG1
Macaques Expressing NIH45-46 & PGT121 IgG1
anti-NIH45-46 IgG1
anti-PGT121 IgG1
anti-3BNC117 IgG1
anti-10-1074 IgG1
A450
Weeks Post AAV Inoculation
124-12
129-12•
228-12
124-12
129-12•
228-12
294-12
329-12
330-12
294-12
329-12
330-12
B
Macaques Expressing 3BNC117 & 10-1074 IgG2
Macaques Expressing NIH45-46 & PGT121 IgG2
anti-NIH45-46 IgG2
anti-PGT121 IgG2
anti-3BNC117 IgG2
anti-10-1074 IgG2
A450
Weeks Post AAV Inoculation
234-12•
240-12•
296-12
234-12•
240-12•
296-12
148-12
173-12•
209-12
148-12
173-12•
209-12

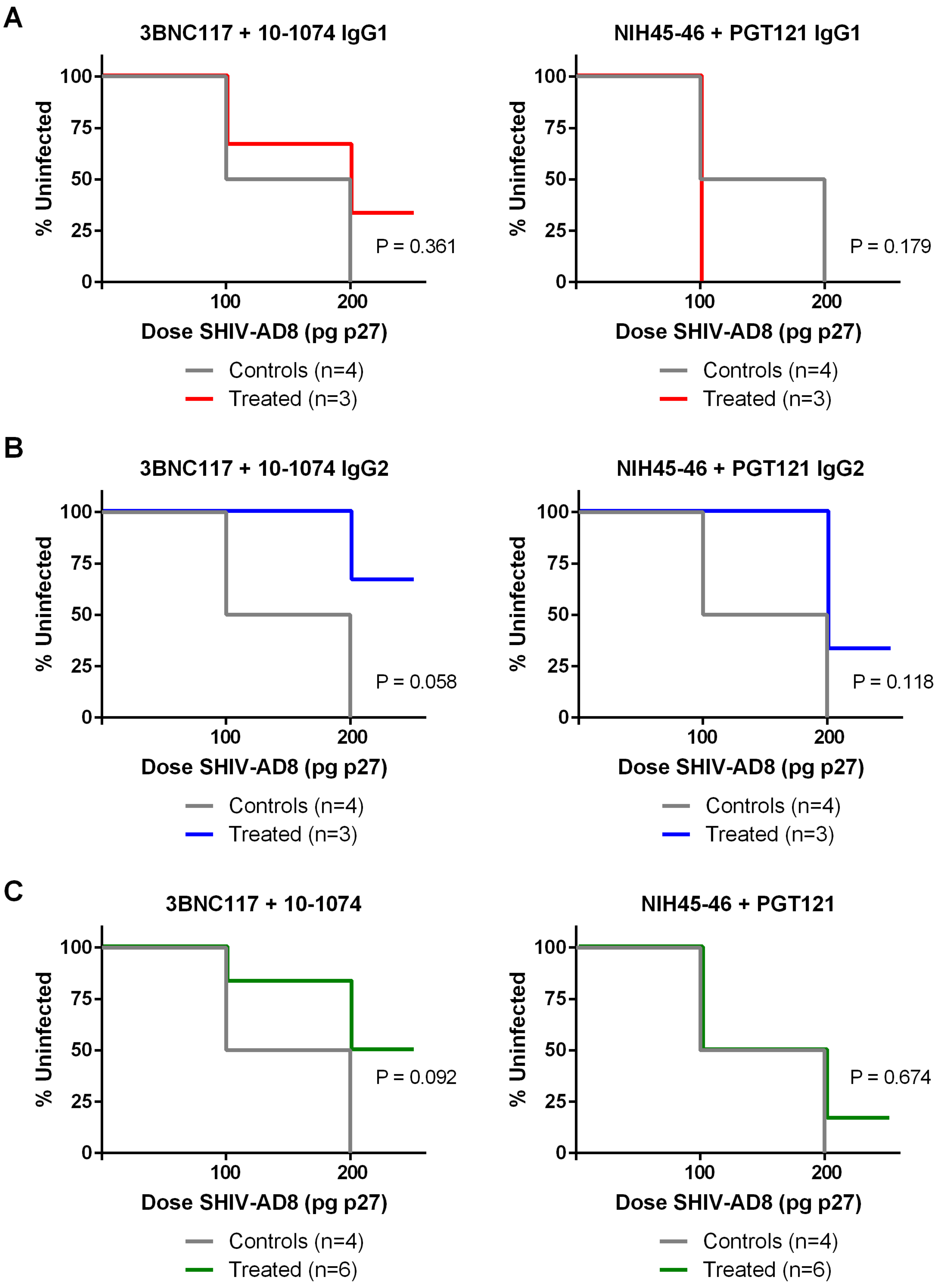
A
3BNC117 + 10-1074 IgG1
% Uninfected
Dose SHIV-AD8 (pg p27)
P = 0.361
Controls (n=4)
Treated (n=3)
NIH45-46 + PGT121 IgG1
% Uninfected
Dose SHIV-AD8 (pg p27)
P = 0.179
Controls (n=4)
Treated (n=3)
B
3BNC117 + 10-1074 IgG2
% Uninfected
Dose SHIV-AD8 (pg p27)
P = 0.058
Controls (n=4)
Treated (n=3)
NIH45-46 + PGT121 IgG2
% Uninfected
Dose SHIV-AD8 (pg p27)
P = 0.118
Controls (n=4)
Treated (n=3)
C
3BNC117 + 10-1074
% Uninfected
Dose SHIV-AD8 (pg p27)
P = 0.092
Controls (n=4)
Treated (n=6)
NIH45-46 + PGT121
% Uninfected
Dose SHIV-AD8 (pg p27)
P = 0.674
Controls (n=4)
Treated (n=6)

Supplemental Figure Legends

Figure S1. Host ADA responses target bNAb variable regions.

(A-B) Assays similar to those described in Figure 4 except that sera diluted 20-fold from macaques expressing IgG1 (A) or IgG2 (B) antibodies were analyzed using plates coated with their IgG2 and IgG1 counterparts, respectively. Values indicate absorbance at 450 nM. Error bars indicate range of measured values. Dots to the right of animal identification indicate protection from SHIV-AD8, as described in Figure 6.

Figure S2. Cross-reactive ADA to bNAbs within the same class.

(A-B) Assays similar to those described in Figure 4 except that sera diluted 20-fold from macaques expressing IgG1 (A) or IgG2 (B) antibodies were analyzed using plates coated with antibodies different from those which the indicated macaques expressed. The sera from animals expressing 3BNC117 and 10-1074 was analyzed with plates coated with NIH45-46 and PGT121 of the same isotype, and vice versa, as indicated. Values indicate absorbance at 450 nM. Error bars indicate range of measured values. Dots to the right of animal identification indicate protection from SHIV-AD8, as described in Figure 6.

Figure S3. Infection curves for macaques expressing individual bNAb pairs.

(A-B) Kaplan-Meier curves indicating infection of control macaques (grey) or macaques expressing indicated IgG1 (A, red) or IgG2 (B, blue) bNAb pairs, with challenge dose indicated on the horizontal axis. (C) Kaplan-Meier curves indicating infection of control macaques (grey) and indicated bNAb pairs regardless of isotype (green). No significant differences were observed in A-C (Mantel-Cox test).
